# Decisional Conflict About Contralateral Prophylactic Mastectomy in Patients with Breast Cancer with and Without Pathogenic Variants in *BRCA* Genes

**DOI:** 10.3390/cancers18061040

**Published:** 2026-03-23

**Authors:** Ji Hyun Sung, Maria C. Katapodi, Sun-Young Park

**Affiliations:** 1 College of Nursing, Keimyung University, Daegu 42601, Republic of Korea; jsung@kmu.ac.kr; 2 Department of Clinical Research, University of Basel, 4055 Basel, Switzerland; maria.katapodi@unibas.ch; 3 College of Nursing, Kyungpook National University, Daegu 41566, Republic of Korea; 4 College of Nursing, Research Institute of Nursing Innovation, Kyungpook National University, Daegu 41566, Republic of Korea

**Keywords:** breast cancer, *BRCA*, decision making, decisional conflict, shared decision making, control preference

## Abstract

**Background/Objectives**: Decision making for contralateral prophylactic mastectomy in patients with breast cancer involves complex risk–benefit trade-offs that may lead to decisional conflict. Understanding factors associated with decisional conflict, particularly for patients with pathogenic variants in *BRCA* genes, is critical for developing tailored decisional support. This study examined decisional conflict, shared decision making, and decisional role preferences in Korean patients with breast cancer considering contralateral prophylactic mastectomy, focusing on factors associated with clinically significant decisional conflict and differences by *BRCA* status. **Methods**: A cross-sectional, internet-based survey was conducted between August and October 2024. Independent *t*-tests, univariate, and multivariate logistic regression analyses identified factors associated with clinically significant decisional conflict. **Results**: The sample included 167 Korean patients with breast cancer (90 *BRCA* carriers and 77 non-carriers). Most patients (76%) experienced clinically significant decisional conflict. Non-carriers reported higher decisional conflict (44.2 vs. 29.3, *p* < 0.001) and lower shared decision making than *BRCA* carriers (44.6 vs. 61.9, *p* < 0.001). Role preferences were similarly distributed across groups (50.3% active, 24.0% collaborative, 25.7% passive). In multivariable analysis, clinically significant decisional conflict in the total sample was associated with non-carrier status (OR = 2.98) and lower shared decision-making scores (OR = 0.94) (*p* < 0.05), explaining 28% of the variance. Among *BRCA* carriers, clinically significant decisional conflict was associated with lower shared decision-making scores (OR = 0.92) and passive role preferences (vs. active) (OR = 4.88). No variables were significantly associated with decisional conflict among non-carriers. **Conclusions**: Findings suggest that decisional conflict is influenced by genetic risk and the quality of the decision-making process. Improving patient engagement by identifying preferred decisional roles, understanding the reasons behind these preferences, and encouraging shared decision making may help reduce decisional conflict, particularly among *BRCA* carriers. Further research is needed to better understand factors associated with decisional conflict among non-carriers.

## Simple Summary

Breast cancer patients may consider contralateral prophylactic mastectomy to reduce the risk of cancer in the opposite breast, especially if they have a genetic predisposition to the disease. However, this complex decision may lead to decisional conflict. This study examined decisional conflict and its associated factors among 167 Korean patients with breast cancer who had genetic testing, comparing differences between patients with and without a pathogenic variant in one of the *BRCA* genes. More than three-quarters of patients experienced clinically significant decisional conflict, particularly non-carriers and those who were less engaged in shared decision making. Among *BRCA* carriers, preference for passive roles in decision making was associated with higher decisional conflict, whereas no significant factors were identified among non-carriers. Findings suggest that decisional conflict is influenced not only by genetic risk but also by the quality of the decision-making process. Strengthening shared decision making and supporting patient engagement may improve decision-making experiences.

## 1. Introduction

Contralateral prophylactic mastectomy involves preventive removal of the healthy breast to reduce risk for contralateral disease in women with unilateral breast cancer [1]. Contralateral prophylactic mastectomy can be considered for women carrying pathogenic variants in the *BRCA1* or *BRCA2* gene [hereafter *BRCA*] or those with a significant family history of breast or ovarian cancer [2]. Decision making regarding contralateral prophylactic mastectomy is a complex trade-off process requiring simultaneous consideration of benefits and risks [1,3,4,5,6]. Patients often struggle to weigh benefits, such as reduced risk for contralateral breast cancer, peace of mind, reduced fear, and survival expectations, against negative consequences including altered body image, decreased sexual function, reduced quality of life, and financial burden [1,3,6,7].

Such complex and preference-sensitive decisions frequently give rise to decisional conflict, defined as a state of uncertainty about a course of action due to insufficient information, unclear personal values, and inadequate decisional support [8,9]. Decisional conflict is commonly assessed using the Decisional Conflict Scale [8,9], which yields scores ranging from 0 to 100 [9], with scores ≥ 25 indicating clinically significant decisional conflict [10,11]. Across various treatment decisions among patients with cancer, high levels of decisional conflict are associated with psychological distress, delays in treatment decisions, and post-decisional regret [11,12]. Accordingly, shared decision making has been emphasized as a key strategy to reduce decisional conflict and improve patient satisfaction with decision outcomes [11,12,13,14,15].

Shared decision making is a collaborative process in which patients and healthcare providers jointly contribute to medical decisions [16,17,18]. In this process, healthcare providers explain treatment options, benefits, and risks, support patients in clarifying their preferences, values, and beliefs, and work with them to make or defer decisions depending on the clinical context [16,17,18,19]. A major challenge to shared decision making is that not all patients wish to actively participate in decision making [3]. Patients’ role preferences are commonly categorized as active (preferring to make decisions independently), collaborative (preferring joint decision making with healthcare providers), or passive (preferring to delegate decisions to healthcare providers) [3,20,21]. Conflicting views exist regarding how healthcare providers should respond to these diverse role preferences, creating a clinical dilemma between respecting patients’ preferred roles and encouraging active participation in decision making.

Prior research suggests that when preferred decision-making roles align with patients’ experiences, decisional conflict and regret decrease, while decisional satisfaction increases [16,22,23]. Similarly, patients’ role preferences have been conceptualized as an expression of autonomy and are therefore consistent with the principles of patient-centered care, which emphasize respect for patient autonomy [16]. Together, these perspectives highlight the importance of tailoring the decision-making process to patients’ stated role preferences.

However, accumulating evidence suggests that active participation in decision making itself may lead to more favorable outcomes, regardless of patients’ initial role preferences. For example, even when patients with prostate cancer [24,25] or lung and colon cancer [22] preferred a passive role, those who actively engaged in the decision-making process and experienced shared decision making reported significantly lower decisional conflict and regret [25], as well as more positive evaluations of care quality and communication [22]. These findings underscore the importance of shared decision making as a means of supporting patient engagement in the decision-making process, independent of patients’ stated role preferences.

Despite this growing body of evidence, little is known about decisional conflict related to contralateral prophylactic mastectomy among Korean patients with breast cancer, including their preferences for engagement in medical decision making and their actual experiences with shared decision making. Moreover, evidence remains limited regarding how pathogenic *BRCA* variant status may influence decisional conflict and shared decision making in this context. The purpose of this study was to describe levels of decisional conflict related to contralateral prophylactic mastectomy among Korean patients with breast cancer, to examine associations with decisional conflict, role preference, and shared decision making, and to compare these associations according to *BRCA* pathogenic variant carrier status.

## 2. Materials and Methods

### 2.1. Study Design and Ethics

This descriptive cross-sectional study used data collected through a self-reported online survey. The study was approved by the Institutional Review Board of the National Evidence-based Healthcare Collaborating Agency, in Korea (IRB No. 24-013-2).

### 2.2. Participants and Recruitment

Eligible participants were women diagnosed with unilateral breast cancer who had genetic testing due to high risk of hereditary breast cancer. Participants were recruited between August and October 2024 through advertisements outlining the eligibility criteria on two online patient platforms: a general breast cancer community (Breast Cancer Story) and a community for patients with triple-negative breast cancer (We Two Beads One).

Individuals who expressed interest in the study completed an initial set of online eligibility questions (Supplementary Materials S1), which assessed whether they met the following inclusion criteria: (1) a diagnosis of breast cancer; (2) age between 20 and 65 years; (3) consideration of or completion of contralateral prophylactic mastectomy; and (4) prior genetic testing based on Korean clinical criteria, including breast cancer diagnosed before the age of 40 years; a first- or second-degree relative diagnosed with breast cancer before 50 years of age, ovarian cancer at any age, or male breast cancer; or a diagnosis of triple-negative breast cancer. Participants were excluded if they reported metastatic (stage IV) disease at initial diagnosis, bilateral breast cancer, or inability to recall whether contralateral prophylactic mastectomy had been considered. Recruitment was not restricted or capped according to pathogenic *BRCA* variant status.

Prior to data collection, eligible participants provided electronic informed consent for study participation and for providing accurate and responsible survey responses (Supplementary Materials S2). Eligible participants completed a self-administered online questionnaire, which required approximately 15–20 min, and received a token of appreciation in the form of a gift voucher valued at KRW 30,000 (approximately USD 20).

Data validation procedures ensured the internal consistency and clinical plausibility of self-reported data. We excluded responses that demonstrated inconsistent tumor subtypes (e.g., estrogen receptor [ER] or progesterone receptor [PR]–positive tumors reported as triple-negative breast cancer), clinically implausible surgical combinations (e.g., breast reconstruction without prior mastectomy), or reports of risk-reducing surgery in the absence of either a strong family history of cancer or a pathogenic variant in the *BRCA* genes.

### 2.3. Measures

#### 2.3.1. Socio-Demographic and Clinical Characteristics 

Participant characteristics included age, marital status, parental status, educational level, household income, employment status, treatment setting (tertiary or general hospital), time since cancer diagnosis, cancer stage at diagnosis, cancer sub-type (hormone receptors, HER2, and triple-negative status), the gene with the pathogenic variant in the *BRCA1* or *BRCA2* gene, whether patients had contralateral prophylactic mastectomy, the type of mastectomy for the affected breast, and family history of cancer.

#### 2.3.2. Dependent Variable

**Decisional conflict.** Decisional conflict was measured using the 16-item Decisional Conflict Scale developed by O’Connor [8], which includes five subscales: informed (3 items), values clarity (3 items), support (3 items), uncertainty (3 items), and effective decision (4 items). Each item was rated on a five-point Likert scale (0 = strongly agree to 4 = strongly disagree). The average of the 16 items was multiplied by 25 to obtain a standardized score ranging from 0–100 scale [9]. Cronbach’s α was 0.95 in this study, comparable to the original report (α = 0.92) [8].

#### 2.3.3. Independent Variables

**Role preference.** Patients’ role preferences in decision making were assessed using the Control Preferences Scale [21]. Five pairs of preferred roles in decision making were presented: A (I prefer to make the final decision); B (I prefer to make the final decision after seriously considering my physician’s opinion); C (I prefer that my physician and I share responsibility for deciding); D (I prefer that my physician makes the final decision but seriously considers my opinion); and E (I prefer to leave all decisions regarding treatment to my physician) [21]. Respondents were classified into three categories: (1) active, indicating a preference for patient-led decision making (A/B); (2) collaborative, indicating a preference for shared decision making (C); and (3) passive, indicating a preference for physician-led decision making (D/E).

**Shared decision making.** Shared decision making regarding contralateral prophylactic mastectomy was assessed using the nine-item Shared Decision-Making Questionnaire (SDM-Q-9) [26]. Examples of items include “the physician clearly explained the benefits and risks of each option,” “the physician asked which option I preferred,” and “the physician and I made the decision about whether to undergo contralateral prophylactic mastectomy together.” Each item was rated on a six-point Likert scale (0 = completely disagree to 5 = completely agree), summed, and converted to a standardized score ranging from 0 to 100 [26]. Higher scores indicate greater participation in shared decision making [26]. Cronbach’s α in this study was 0.94, consistent with the original validation [26].

**Translation of scales.** All scales were translated into Korean by a member of the research team and adapted to reflect the clinical context of the target population. Linguistic accuracy was assessed by a native Korean–English bilingual speaker. The clinical relevance of the translated scales for Korean patients with breast cancer was evaluated by four Korean advanced practice nurses working in tertiary hospitals or cancer centers and two nursing professors with expertise in questionnaire development.

### 2.4. Statistical Analyses

Power analysis, calculated using G*Power Version 3.1.9.2 (University of Düsseldorf, Düsseldorf, Germany) [27], with an alpha level of 0.05, power of 0.95, an expected incidence rate of clinically significantly decisional conflict of 0.50, and a moderate effect size corresponding to an odds ratio (OR) of 2.0 for the association between decisional conflict and preference for involvement in healthcare decisions [28], indicated a required sample of 129 participants. Thus, the final sample of 167 participants exceeded the required minimum.

Statistical analyses were performed using R software (version 4.4.1; R Foundation for Statistical Computing, Vienna, Austria), with the level of significance set at *p* < 0.05. Descriptive statistics (means, standard deviations [SDs], frequencies, and percentages) were used to summarize participants’ socio-demographic and clinical characteristics, such as levels on the decisional conflict scale, shared decision making, and role preferences. Group differences between patients with and without pathogenic variants in *BRCA* genes were examined using independent *t*-tests for continuous variables and Pearson’s chi-square tests for categorical variables.

Univariate logistic regression analyses were performed to identify socio-demographic and clinical factors, and independent variables associated with clinically significant decisional conflict. Multivariable logistic regression was then conducted to examine the influence of shared decision making and role preference on clinically significant decisional conflict in the context of contralateral prophylactic mastectomy. Variables showing significant associations in univariate analyses and those considered clinically relevant were included in the multivariable model to adjust for potential confounders associated with decisional conflict. Sub-group analyses stratified by pathogenic *BRCA* status were performed. Following Harrell’s guideline of 10–20 events per independent variable for logistic regression models [29], a maximum of seven independent variables was included in the multivariable logistic regression analysis, based on the smallest sub-group size (*n* = 77). ORs with 95% confidence intervals (CIs) were reported to indicate the strength and precision of the associations.

## 3. Results

### 3.1. Participant Characteristics

Of the 200 participants who completed the self-administered online questionnaire, 29 with unknown genetic testing results and four with pathogenic variants in other genes (e.g., *PALB2*, *TP53*) were excluded, yielding a final analytic sample of 167 individuals.

Among 167 participants, 55.9% (*n* = 90) were carriers of pathogenic variants in one of the *BRCA* genes [56.7% (*n* = 51) *BRCA1* and 43.3% (*n* = 39) *BRCA2*]. Patients with pathogenic variants were younger (mean = 42.7 years, SD = 7.9) compared to those without (mean = 46.5 years, SD = 8.5). Most participants were married (80.2%), had children (79.6%), and held a college or university degree or higher (84.5%). Over half (52.1%) were employed, 37.7% reported a monthly income exceeding USD 3500 (the average monthly household income in Korean was USD 2920 in 2024) (Table 1).

Most patients (80.8%) underwent breast cancer surgery at tertiary hospitals and 19.2% were treated at general hospitals. The mean time since cancer diagnosis was 38.9 months (SD = 37.2). Cancer stage at diagnosis was stage II in 47.3%, stage I in 22.8%, and stage III in 27.5%. Triple-negative breast cancer was the most common cancer subtype (74.3%), followed by ER/PR-positive (25.7%) and HER2-positive (4.2%) tumors. In total, 96 patients (57.5%) underwent total mastectomy for the affected breast, 55 patients (32.9%) underwent contralateral prophylactic mastectomy, and 70.0% reported family history of cancer.

### 3.2. Decisional Conflict, Shared Decision Making, and Role Preferences

Table 2 shows that patients without a pathogenic *BRCA* variant had significantly higher decisional conflict scores than those with pathogenic variants across both total scores (44.2 vs. 29.3; t = 5.23, *p* < 0.001) and all subscales, including informed, values clarity, support, uncertainty, and effective decision (all *p* < 0.001). Clinically significant decisional conflict was prevalent in most participants (76.0%), with a significantly higher proportion among patients without pathogenic *BRCA* variants (89.6% vs. 64.5%; *p* < 0.001), indicating that this sub-group constitutes an at-risk population for heightened decisional conflict.

Among 167 participants, approximately half preferred an active role in decision making (50.3%), followed by collaborative (24.0%) and passive roles (25.7%), with no significant differences between *BRCA* status groups (χ^2^ = 0.33, *p* = 0.846).

Patients without pathogenic *BRCA* variants reported significantly lower levels of shared decision making compared to those with pathogenic variants (44.6 vs. 61.9; *t* = –4.85, *p* < 0.001), suggesting reduced perceived involvement in the decision-making process within this at-risk group.

### 3.3. Factors Associated with Decisional Conflict (Univariate Logistic Regression)

Table 3 presents univariate logistic regression results for factors associated with clinically significant decisional conflict in the entire sample and by pathogenic *BRCA* status. In the entire sample, higher odds of having clinically significant decisional conflict were significantly associated with older age (OR = 1.05, 95% CI: 1.01–1.11), not undergoing contralateral prophylactic mastectomy (OR = 3.45, 95% CI: 1.54–7.14), undergoing partial mastectomy of the affected breast (vs. total mastectomy) (OR = 2.29, 95% CI: 1.07–4.94), and lower levels of shared decision making (OR = 0.92, 95% CI: 0.89–0.96).

Among patients with *BRCA* pathogenic variants, clinically significant decisional conflict was significantly associated with lower shared decision-making scores (OR = 0.93, 95% CI: 0.88–0.98) and a preference for a passive role compared to an active role (OR = 4.20, 95% CI: 1.24–14.20). No significant difference was observed between active and collaborative role preferences (OR = 1.46, 95% CI: 0.61–3.51). In patients without pathogenic variants, no variables were significantly associated with clinically significant decisional conflict.

### 3.4. Factors Associated with Decisional Conflict (Multivariate Logistic Regression)

Table 4 presents the results of the multivariate logistic regression for the entire sample, showing that a higher likelihood of clinically significant decisional conflict was associated with not carrying a pathogenic *BRCA* variant, compared to carrying one (OR = 2.98, 95% CI: 1.02–8.72), and lower shared decision-making scores (OR = 0.94, 95% CI: 0.90–0.98). The model explained 28% of the variance in decisional conflict (Nagelkerke R^2^ = 0.28, *p* < 0.001).

Sub-group analyses by *BRCA* status showed that among patients with pathogenic *BRCA* variants, lower shared decision-making scores (OR = 0.92, 95% CI: 0.87–0.98, *p* = 0.006) and a passive role preference (vs. active) (OR = 4.88, 95% CI: 1.25–19.01, *p* = 0.022) were significantly associated with higher odds of clinically significant decisional conflict, while collaborative role preference showed no significant association (OR = 2.02, 95% CI: 0.62–6.62, *p* = 0.244). The model explained 25% of the variance (Nagelkerke R^2^ = 0.25, *p* < 0.001). In contrast, among patients without pathogenic variants, none of the variables were significantly associated with clinically significant decisional conflict. The model was not statistically significant in this sub-group (Nagelkerke R^2^ = 0.18, *p* = 0.447).

## 4. Discussion

This study examined decisional conflict, shared decision making, and role preferences in Korean patients with breast cancer considering contralateral prophylactic mastectomy, focusing on factors associated with decisional conflict and differences by pathogenic *BRCA* status. Over three-quarters of patients had clinically significant decisional conflict. Patients without pathogenic *BRCA* variants reported higher decisional conflict and lower shared decision-making engagement compared with their counterparts. Clinically significant decisional conflict among patients with pathogenic *BRCA* variants was associated with lower shared decision making and passive role preference, whereas no significant associated factors were identified among patients without pathogenic *BRCA* variants.

The high prevalence of clinically significant decisional conflict suggests that decisions about contralateral prophylactic mastectomy demand complex evaluations under conditions of uncertainty [1,6,30]. The mean score of decisional conflict among patients with pathogenic *BRCA* variants was 29.3, which is consistent with previously reported ranges (21–38) in similar samples from Australia [31], Canada [1], and Germany [32].

In contrast, the majority of patients (89.6%) without a pathogenic *BRCA* variant experienced clinically significant decisional conflict, with a mean score of 44.2—substantially higher than the mean score of 26.7 reported in a comparable Canadian sample, in which fewer than 27% exhibited clinically significant decisional conflict [4]. This finding indicates a markedly elevated level of decisional conflict among Korean women without pathogenic *BRCA* variants. Notably, decisional conflict was significantly higher among patients without a pathogenic *BRCA* variant than among carriers, contrary to prior assumptions that carriers of pathogenic variants in *BRCA* genes would experience greater decisional conflict regarding contralateral prophylactic mastectomy [1,4,6,32]. The overall pattern of decisions regarding contralateral prophylactic mastectomy appeared consistent with current recommendations of clinical guidelines (e.g., the National Comprehensive Cancer Network Guidelines and the American Society of Breast Surgeons Guidelines) [2,33], with most patients with pathogenic *BRCA* variants, and relatively few non-carriers, undergoing contralateral prophylactic mastectomy. However, clinically appropriate decisions did not necessarily translate into satisfactory decision-making experiences, particularly among patients without a pathogenic variant. Study results showed that even when decisions align with guideline-based risk management strategies, patients may still experience uncertainty, emotional distress, or unmet informational needs during the decision-making process. These findings suggest potential gaps in post-test genetic counseling for patients receiving negative genetic testing results, including insufficient information provision and inadequate clarification of individualized risk and management options [8,9]. Strengthening post-test counseling for non-carriers may therefore help improve decision-making experiences.

Notably, although decisional conflict was markedly higher among patients without a pathogenic variant, none of the examined variables were significantly associated with decisional conflict in this sub-group. This result may partly reflect limited statistical power due to the small sample size (*n* = 77). However, it may also indicate differences in the counseling and decision-support context for patients without a pathogenic variant, for whom contralateral prophylactic mastectomy is generally not recommended in international clinical guidelines [2]. Previous studies have shown that approximately 30–39.3% of patients at average genetic risk reported no substantial discussion about contralateral prophylactic mastectomy with their physicians [34,35]. In Korea, access to genetic counseling remains limited because counseling services are not covered by the national health insurance system, even for patients with a pathogenic *BRCA* variant [6]. Under such conditions, counseling for patients without a pathogenic variant may occur even less frequently, further limiting opportunities for shared decision making and discussion of preventive surgical options. Consequently, patients in this sub-group may have fewer opportunities to engage in shared decision making or clarify their preferred roles in the decision-making process, which may explain why decision-process variables, such as shared decision making and role preference, were not significantly associated with decisional conflict in this group. Prior studies suggest that cognitive and psychological factors, including perceived benefits of preventive surgery [4,36], decisional self-efficacy [4], and perceived contralateral breast cancer risk [36], may play a more prominent role in shaping decisional conflict among women without pathogenic *BRCA* variants.

Taken together, our findings regarding decisional conflict in patients without pathogenic *BRCA* variants suggest the need for tailored decisional support and structured, evidence-based counseling for patients with uninformative negative genetic testing results. In addition, these findings indicate the need for further research with larger samples to identify factors influencing decisional conflict among patients without pathogenic variants. Future studies should also examine the availability of genetic counseling for this population and its influence on decisional conflict.

Shared decision-making scores showed a clear inverse association with clinically significant decisional conflict, consistent with prior findings [19,37]. This finding adds to a growing body of evidence indicating that shared decision making is associated with favorable patient outcomes across various clinical contexts, including reduced decisional conflict [3], improved satisfaction with care [3,35], a stronger patient–clinician relationship [17], and greater patient self-advocacy in healthcare management [4]. These findings, consistent with prior research, reinforce the importance of strengthening shared decision making when considering contralateral prophylactic mastectomy, particularly in contexts where patients may have limited engagement in the decision-making process.

Approximately one-quarter of the sample preferred a passive role when deciding on contralateral prophylactic mastectomy, consistent with other studies [10,11,14,38,39]. In the present study, sub-group analyses further showed that among patients with pathogenic *BRCA* variants, those who preferred a passive role were more likely to experience higher decisional conflict. These findings are noteworthy because they challenge the assumption that passive role preferences necessarily reflect autonomous and stable decision-making styles [16] and that concordance between preferred and actual decision-making roles consistently optimizes decisional outcomes such as decisional conflict and regret [16,22,23]. Rather, our findings align with prior studies suggesting that greater patient involvement in decision making is often associated with lower decisional conflict [22,24,25]. Alternatively, passive preferences may reflect insufficient information, unclear values regarding treatment options, or a desire for guidance from trusted clinicians in complex and emotionally challenging situations.

Taken together, the observed associations suggest that decisional conflict may be influenced not only by pathogenic variant status but also by the quality of the decision-making process, including the level of shared decision making and the formation of patients’ role preferences. These findings highlight the need for approaches that promote patient-centered decision making and help alleviate decisional conflict. First, identifying patients’ preferred roles in the decision-making process is essential. When patients prefer a passive role, it is important to explore the reasons underlying this preference and provide tailored support to facilitate more engagement in decision making. Such support may include providing clear and relevant information, offering emotional support, and facilitating values clarification to help patients articulate their priorities and concerns [4,16,22,25]. Through these processes, patients may strengthen their decisional self-efficacy [4,16,22,25], which in turn may help reduce decisional conflict.

Second, shared decision making may be beneficial for all patients considering contralateral prophylactic mastectomy, regardless of pathogenic variant carrier status. This recommendation is particularly relevant in Asian clinical settings, such as Korea, where patient participation is often limited by hierarchical or paternalistic patient–clinician relationships [6,40,41], and shared decision making remains insufficiently established [42]. In such clinical environments, a multi-level approach may be helpful to promote more patient-centered decision making and alleviate decisional conflict. Specifically, embedding shared decision making into clinical care processes systematically and structurally [18], expanding the use of brief decision aids to provide the resources necessary for implementing shared decision making [3,10], and strengthening healthcare providers’ clinical communication competencies [19] may help strengthen shared decision making.

This study has several limitations. First, participants were recruited through online communities of patients with (triple-negative) breast cancer, which may have introduced selection bias. Individuals who actively participate in online patient communities may have higher health literacy and greater engagement in healthcare services. Therefore, findings may not be fully generalizable to the broader population of patients undergoing genetic testing and considering contralateral prophylactic mastectomy. Second, the cross-sectional measurement of decisional conflict at a single time point limits the ability to capture its dynamic nature throughout the decision-making process. As participants completed the survey approximately 40 months after diagnosis, the varying time intervals between initial consideration of surgery and survey completion may have introduced recall bias. Third, the cross-sectional design of this study limits the ability to establish causal relationships among variables. Accordingly, the observed associations with decisional conflict, shared decision making, and role preference should not be interpreted as directional. Future prospective longitudinal studies are needed to examine temporal changes in decisional conflict and to clarify the directionality of relationships. Finally, several odds ratio estimates showed relatively wide confidence intervals, indicating limited precision. These findings should be interpreted with caution, particularly in sub-group analyses with smaller sample sizes. Future studies with larger samples are needed to obtain more precise estimates of the associations identified in this study.

## 5. Conclusions

This study identified substantial decisional conflict in Korean patients with breast cancer considering contralateral prophylactic mastectomy, particularly among those without a pathogenic *BRCA* variant and those who reported lower levels of engagement in shared decision making. Among patients with pathogenic variants, lower shared decision-making scores and passive role preference were associated with greater decisional conflict. No significant associated factors were identified among those without pathogenic variants. The findings suggest that decisional conflict is shaped not only by pathogenic variant status but also by the quality of the decision-making process. Strengthening shared decision making tailored to *BRCA* status and supporting patient engagement in the decision-making process, by identifying patients’ preferred roles, understanding the reasons behind these preferences, and encouraging participation, may help reduce decisional conflict, particularly among patients with pathogenic variants. Further research is needed to better understand factors associated with decisional conflict among non-carriers.

## Figures and Tables

**Table 1:** Characteristics of patients with and without a pathogenic variant in *BRCA* genes.

Variables		Total (*n* = 167)	*BRCA* (+) (*n* = 90, 55.9%)	*BRCA* (−) (*n* = 77, 47.8%)	t/χ^2^	*p*
		*n* (%) or Mean ± SD				
**Socio-demographic characteristic**						
Age (years)		44.5 ± 8.3	42.7 ± 7.9	46.5 ± 8.5	2.99	0.003
Marital status	Single/separated/divorced/widowed	33 (19.8)	21 (23.3)	12 (15.6)	1.57	0.210
	Married/living as married	134 (80.2)	69 (76.7)	65 (84.4)		
Having children	No	34 (20.4)	20 (22.2)	14 (18.2)	0.42	0.518
	Yes	133 (79.6)	70 (77.8)	63 (81.8)		
Education	High school graduate	26 (15.6)	11 (12.2)	15 (19.5)	5.66	0.059
	College/university degree	117 (70.1)	70 (77.8)	47 (61.0)		
	Master/PhD	24 (14.4)	9 (10.0)	15 (19.5)		
Household income (USD/month)	≤3500	104 (62.3)	57 (63.3)	47 (61.0)	0.09	0.760
	>3500	63 (37.7)	33 (36.7)	30 (39.0)		
Employed	No	80 (47.9)	42 (46.7)	38 (49.4)	0.12	0.729
	Yes	87 (52.1)	48 (53.3)	39 (50.6)		
**Clinical characteristic**						
Treatment setting	Tertiary hospital	135 (80.8)	72 (80.0)	63 (81.8)	0.09	0.766
	General hospital	32 (19.2)	18 (20.0)	14 (18.2)		
Time since cancer diagnosis (months)		38.9 ± 37.2	36.8 ± 35.5	41.32 ± 39.2	0.775	0.439
Cancer stage at diagnosis	I	38 (22.8)	21 (23.3)	17 (22.1)	10.19	0.017
	II	79 (47.3)	35 (38.9)	44 (57.1)		
	III	46 (27.5)	33 (36.7)	13 (16.9)		
	Unknown	4 (2.4)	1 (1.1)	3 (3.9)		
Cancer subtype	ER or PR positive	43 (25.7)	30 (33.3)	13 (16.9)	5.64	0.018
	HER2 positive	7 (4.2)	1 (1.1)	6 (7.8)	4.71	0.030
	TNBC	124 (74.3)	60 (66.6)	63 (81.8)	5.28	0.022
Type of mastectomy for the affected breast	Total mastectomy	96 (57.5)	66 (73.3)	30 (39.0)	20.27	<0.001
	Partial mastectomy	69 (41.3)	23 (25.6)	46 (59.7)		
Pathogenic variant	*BRCA1*		51 (56.7)			
	*BRCA2*		39 (43.3)			
Had contralateral prophylactic mastectomy	Yes	55 (32.9)	52 (57.8)	3 (3.9)	57.44	<0.001
Had family history of cancer	Yes	117 (70.1)	72 (80.0)	45 (58.4)	9.20	0.002

ER, estrogen receptor; HER2, human epidermal growth factor receptor 2; PR, progesterone receptor; SD, standard deviation; TNBC, triple-negative breast cancer. USD 1 = KRW 1430 (October 2024).

**Table 2:** Decisional conflict, shared decision making, and role preferences of patients with and without a pathogenic variant in *BRCA* genes.

Variable and Sub-Scales	Total (*n* = 167)	*BRCA* (+) (*n* = 90)	*BRCA* (−) (*n* = 77)	t/χ^2^	*p*
	Mean ± SD or No. of Patients (%)				
**Decisional conflict scale**					
Total score	36.2 ± 19.6	29.3 ± 18.3	44.2 ± 17.9	5.23	<0.001
Informed	34.2 ± 22.2	27.3 ± 20.1	42.2 ± 21.9	4.56	<0.001
Values clarity	37.2 ± 24.4	29.6 ± 22.5	46.1 ± 23.7	4.61	<0.001
Support	37.6 ± 20.4	31.2 ± 19.7	45.0 ± 18.8	4.62	<0.001
Uncertainty	38.8 ± 24.1	30.8 ± 22.6	48.2 ± 22.5	4.95	<0.001
Effective decision	33.9 ± 22.2	28.1 ± 22.1	40.7 ± 20.5	3.83	<0.001
**Clinically significant decisional conflict**					
No	40 (24.0)	32 (35.5)	8 (10.4)	14.43	<0.001
Yes	127 (76.0)	58 (64.5)	69 (89.6)		
**Shared decision making**					
Total score	56.9 ± 24.4	61.9 ± 21.4	44.6 ± 24.6	−4.85	<0.001
**Role preferences**					
Active, patient decides	84 (50.3)	46 (51.1)	38 (49.3)	0.33	0.846
Collaborative, shared decision	40 (24.0)	20 (22.2)	20 (26.0)		
Passive, physician decides	43 (25.7)	24 (26.7)	19 (24.7)		

SD, standard deviation.

**Table 3:** Univariate logistic regression of clinically significant decisional conflict.

Variables		Clinically Significant Decisional Conflict OR (95% CI)		
		Total (*n* = 167)	*BRCA* (+) (*n* = 90)	*BRCA* (−) (*n* = 77)
**Socio-demographic variables**				
Age		1.05 (1.01–1.10) *	1.05 (0.99–1.11)	1.00 (0.91–1.09)
Marital status (ref: single/separated/divorced/widowed)	Married	1.81 (0.79–4.16)	1.50 (0.55–4.07)	1.97 (0.35–11.15)
Having children (ref: no)	Yes	1.43 (0.62–3.32)	1.67 (0.61–4.6)	0.62 (0.07–5.45)
Education		0.85 (0.44–1.63)	0.68 (0.27–1.74)	1.00 (0.31–3.23)
Household income		0.94 (0.72–1.24)	0.99 (0.72–1.34)	0.57 (0.26–1.24)
Employed (ref: no)	Yes	1.02 (0.50–2.08)	1.20 (0.50–2.86)	0.55 (0.12–2.48)
**Clinical variables**				
Treatment setting (ref: tertiary hospital)	General hospital	2.54 (0.84–7.76)	2.23 (0.67–7.45)	Not estimable
Time since cancer diagnosis		1.00 (0.99–1.01)	1.00 (0.99–1.01)	0.99 (0.97–1.00)
Cancer stage at the diagnosis		0.99 (0.65–1.51)	1.18 (0.70–2.02)	0.78 (0.36–1.68)
ER or PR positive (ref: negative)	Yes	1.10 (0.75–1.62)	1.19 (0.66–2.14)	1.11 (0.38–3.2)
HER2 positive (ref: negative)	Yes	2.05 (0.29–14.24)	NA	1.12 (0.51–2.44)
Triple-negative breast cancer (ref: no)	Yes	1.27 (0.75–2.16)	1.11 (0.65–1.90)	1.19 (0.46–3.11)
Type of pathogenic variant (ref: *BRCA1*)	*BRCA2*		1.78 (0.73–4.35)	
Had contralateral prophylactic mastectomy (ref: yes)	No	3.45 (1.54–7.14) *	0.60 (0.25–1.47)	0.21 (0.02–2.61)
Type of mastectomy for the affected breast (ref: total mastectomy)	Partial mastectomy	2.29 (1.07–4.94) *	1.89 (0.70–5.08)	1.11 (0.38–3.20)
Having familiar cancer history	Yes	0.72 (0.32–1.63)	0.45 (0.13–1.50)	4.96 (0.93–26.43)
**Independent variables**				
Shared decision making		0.92 (0.89–0.96) *	0.93 (0.88–0.98) *	0.95 (0.89–1.02)
Role preferences (ref: active, patient decides)	Collaborative, shared decision	1.46 (0.61–3.51)	1.56 (0.53–4.62)	0.06 (0.18–6.34)
	Passive, physician decides	2.61 (0.98–6.97)	4.20 (1.24–14.2) *	1.00 (0.17–6.02)

* *p* < 0.05 CI, confidence interval; ER, estrogen receptor; HER2, human epidermal growth factor receptor 2; NA, not applicable; OR, odds ratio; PR, progesterone receptor; ref, reference group.

**Table 4:** Multivariate logistic regression of clinically significant decisional conflict.

Variables		Clinically Significant Decisional Conflict					
		Total (*n* = 167)		*BRCA* (+) (*n* = 90)		*BRCA* (−) (*n* = 77)	
		OR (95% CI)	*p*	OR (95% CI)	*p*	OR (95% CI)	*p*
Patient *BRCA* (−) (ref: patient *BRCA* (+))		2.98 (1.02–8.72)	0.046	-		-	
Age		1.02 (0.97–1.07)	0.491	1.02 (0.96–1.09)	0.530	0.99 (0.90–1.09)	0.855
Treatment setting (ref: tertiary hospital)	General hospital	2.78 (0.82–9.40)	0.101	1.86 (0.47–7.40)	0.381	Not estimable	0.999
Had contralateral prophylactic mastectomy (ref: yes)	No	1.20 (0.37–3.87)	0.760	0.68 (0.17–2.82)	0.599	5.02 (0.27–91.8)	0.276
Type of mastectomy for the affected breast (ref: total mastectomy)	Partial mastectomy	0.98 (0.31–3.05)	0.966	1.76 (0.32–9.62)	0.516	0.69 (0.11–4.15)	0.684
Shared decision making		0.94 (0.90–0.98)	0.003	0.92 (0.87–0.98)	0.006	0.95 (0.88–1.02)	0.165
Role preferences (ref: active, patient decides)	Collaborative, shared decision	1.66 (0.62–4.44)	0.312	2.02 (0.62–6.62)	0.244	1.29 (0.20–8.36)	0.788
	Passive, physician decides	2.95 (0.99–8.77)	0.052	4.88 (1.25–19.01)	0.022	0.99 (0.14–7.00)	0.988
**Model**							
R^2^ (Nagelkerke)		0.28	<0.001	0.25	0.013	0.18	0.447
Hosmer–Lemeshow goodness-of-fit (χ^2^)		14.04	0.081	10.58	0.226	10.80	0.213

CI, confidence interval; OR, odds ratio; ref, reference group.

## Data Availability

The original contributions presented in this study are included in the article/Supplementary Materials.

## Supplementary Materials

The following supporting information can be downloaded at: https://www.mdpi.com/article/10.3390/cancers18061040/s1, Supplementary Materials S1. Eligibility Screening Questions. Supplementary Materials S2. Informed Consent for Honest and Responsible Survey Participation
