# Supplementary material for: Decisional Conflict About Contralateral Prophylactic Mastectomy in Patients with Breast Cancer with and Without Pathogenic Variants in BRCA Genes
Source: Cancers (Basel). 2026 Mar 23;18(6):1040. doi: 10.3390/cancers18061040 (PMC13024840; doi:10.3390/cancers18061040)
Supplement: Supplementary file 1 [file cancers-18-01040-s001.zip › cancers-4170120-supplementary.pdf]

Supplementary Material S1. Eligibility Screening Questions

1. Basic eligibility criteria

Please indicate whether you meet all of the following criteria.

· I am a woman diagnosed with breast cancer.

· I am between 20 and 65 years of age.

· I have previously considered contralateral prophylactic mastectomy (CPM).

☐ Yes, I meet all three criteria

☐ No, I do not meet at least one of the criteria

Screening process: Participants who do not meet all three criteria will be excluded.

2. Genetic testing experience

Have you ever undergone *BRCA*1/2 genetic testing for hereditary breast cancer?

☐ Yes

☐ No

☐ Not sure

Screening process: Participants who select “No” or “Not sure” was excluded.

3. Hereditary breast cancer–related risk factors

Please select all that apply to you. *(Multiple responses allowed)*

☐ Diagnosed with breasts cancer before 40 years old

☐ At least one first-degree family member (parent, sibling, or child) diagnosed with ovarian cancer, breast cancer before age 50, or male breast cancer

☐ Diagnosed with triple-negative breast cancer

☐ Diagnosed with multicentric or multifocal breast cancer

☐ Recommended contralateral mastectomy by a healthcare professional

☐ None of the above

Screening process: Participants who select “None of the above” were excluded.

4. Cancer status

Have you ever been diagnosed with metastatic (stage IV) breast cancer at the time of initial diagnosis??

☐ Yes

☐ No

☐ Not sure

Screening process: Participants who select "Yes" was excluded.

5. Contralateral prophylactic mastectomy timing-related screening

At what point did you consider contralateral prophylactic mastectomy?

☐ At the time of initial breast cancer diagnosis
☐ During treatment planning (before surgery)
☐ After initial surgery
☐ I do not remember / Not sure

Screening process: Participants who select "I do not remember / Not sure" was excluded.

Supplementary Material S2. Informed Consent for Honest and Responsible Survey Participation

Providing false information or submitting duplicate responses is considered a serious violation of research ethics.

If intentional false or duplicate responses are identified, the participant will be excluded from the study, all submitted data will be discarded, and compensation will not be provided.

Such responses may also compromise the integrity of the study and negatively affect the validity of other participants' data. Therefore, participants are expected to take full responsibility for honest and single participation.

You may participate in this survey only if you agree to both statements below.

☐ I understand that false or duplicate responses constitute a serious ethical violation and may result in exclusion from the study and loss of compensation.

☐ I agree to participate honestly and submit only one response.
